# Size‐Controlled Graphene Nanodot Arrays/ZnO Hybrids for High‐Performance UV Photodetectors

**DOI:** 10.1002/advs.201700334

**Published:** 2017-11-17

**Authors:** Ruidie Tang, Sancan Han, Feng Teng, Kai Hu, Zhiming Zhang, Mingxiang Hu, Xiaosheng Fang

**Affiliations:** ^1^ Department of Materials Science Fudan University Shanghai 200433 P. R. China; ^2^ Department of Materials Science and Engineering University of Shanghai for Science and Technology Shanghai 200093 P. R. China

**Keywords:** graphene nanodots arrays, lithography, photodetectors, ZnO

## Abstract

Graphene nanodots (GNDs) are one of the most attractive graphene nanostructures due to their tunable optoelectronic properties. Fabricated by polystyrene‐nanosphere lithography, uniformly sized graphene nanodots array (GNDA) is constructed as an ultraviolet photodetector (PD) with ZnO nanofilm spin coated on it. The size of GNDA can be well controlled from 45 to 20 nm varying the etching time. It is revealed in the study that the photoelectric properties of ZnO/GNDA PD are highly GNDA size‐dependent. The highest responsivity (*R*) and external quantum efficiency of ZnO/GNDA (20 nm) PD are 22.55 mA W^−1^ and 9.32%, almost twofold of that of ZnO PD. Both ZnO/GNDA (20 nm) PD and ZnO/GNDA (30 nm) PD exhibit much faster response speed under on/off switching light and have shorter rise/decay time compared with ZnO PD. However, as the size of GNDA increase to 45 nm, the PD appears poor performance. The size‐dependent phenomenon can be explained by the energy band alignments in ZnO/GNDA hybrids. These efforts reveal the enhancement of GNDs on traditional photodetectors with tunable optoelectronic properties and hold great potential to pave a new way to explore the various remarkable photodetection performances by controlling the size of the nanostructures.

## 1 Introduction

Graphene has attracted much attention because of its unique superiority in high carrier mobility, conductivity, transparency, chemical stability, mechanical strength, and high elasticity.^1^ However, the zero‐band gap nature hinders its applications in optoelectronic devices.^2^ Many researches have been conducted to open the band gap by fabricating nanostructures such as graphene nanodots (GNDs),^3^ graphene nanomeshes,^4^ and graphene nanoribbons.^5^ Compared with graphene, GNDs have excellent optical and electrical properties, which are tunable depending on size and shape,^6^ thus holding promising application prospects in photoelectric devices.^7^

**Scheme 1:**
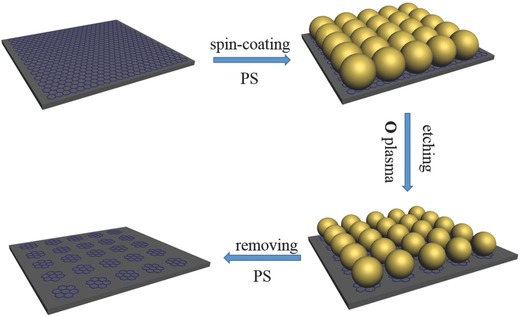
Schematic illustration of the fabrication procedures of the GNDA based on PS‐NS lithography. First, a closely packed PS NSs layer was assembled on the graphene/silicon wafer substrates by spin‐coating. Then RIE was employed to fabricate graphene nanodots array with PS NS layer as mask. During RIE, PS NSs and graphene unprotected by PS NSs were etched by oxygen plasma. As a result, only graphene underneath the remaining PS NSs was left. Finanlly, the remaining PS NSs were removed selectively, and the orderly aligned GNDA were obtained.

The optoelectronic properties of GNDs are highly size dependable^8^ and it has been demonstrated that patterning periodic structures will influence the optical fields in graphene,^9^ so it is critical to control the size and alignment of GNDs for exploring the graphene nanodots‐based electro‐optical devices. Many methods have been tried to fabricate GNDs, such as Hummers method,^10^ hydrothermal process,^11^ and electrochemical method,^12^ but it is difficult to accurately control the size and orderly alignment of GNDs using these methods. Here lithography based on reactive ion etching (RIE) with polystyrene (PS)‐nanosphere (NS) as template is employed to fabricate uniformly sized graphene nanodots array (GNDA) directly from large‐scale graphene as illustrated in **Scheme** **1**. The size of GNDA could be controlled by O_2_‐plasma etching time.

**Scheme 2:**
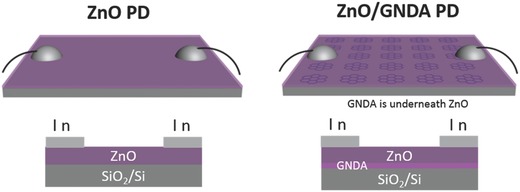
Schematic illustration of fabricated ZnO photodetectors and ZnO/GNDA photodetectors.

UV photodetectors (PDs) have great value and a wide range of applications, including communications, flame detection, optical imaging, ozone hole monitoring, and air and water sterilization.^13^ Among the variety of materials, ZnO as an environmentally friendly semiconductor has drawn great attention for UV detector because of its wide band gap (≈3.37 eV at room temperature), low cost, and easy fabrication.^14^ It is reported that monolayer graphene film^15^ and reduced graphene oxide^16^ have been introduced to combine with ZnO nanostructures to enhance performance ZnO‐based photodetectors. But as to our knowledge, there is no reported research that reveals the function of GNDA in photodetector.^16^, ^17^ In order to explore the application potential of GNDA in photodetector, GNDA was combined with ZnO nanofilm to fabricate ZnO/GNDA hybrids UV photodetector, of which the performance were compared with ZnO photodetector (**Scheme** **2**). The photodetection performance of ZnO/GNDA PD was highly GNDA size‐dependent. ZnO/GNDA (20 nm) PD and ZnO/GNDA (30 nm) PD exhibited better performance than ZnO PD. However, the ZnO/GNDA (45 nm) PD appeared worse performance, compared with ZnO PD. The energy band alignments in ZnO/GNDA composites could be used to explain the size‐dependent phenomenon. These efforts broaden the application potential of GNDA in photodetectors and pave a new way to explore the various photodetection performances by controlling the size of the nanostructures.

## 2 Results and Discussion

### 2.1 Characterization of Graphene Nanodots Arrays

**Figure 1:**
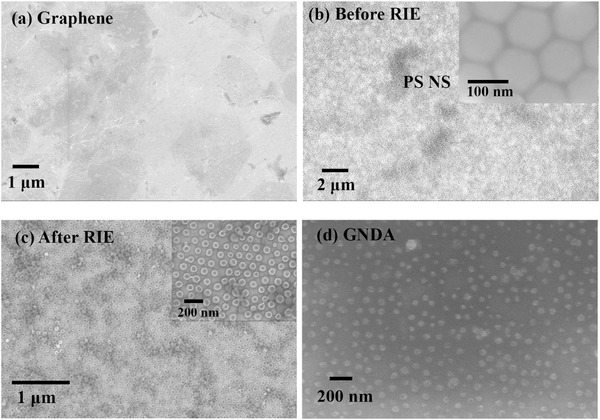
a) SEM image of purchased graphene on an SiO_2_/Si substrate. b) SEM image of PS NSs layer on graphene on SiO_2_/Si substrate. c) PS‐NS arrays reduced after O_2_‐plasma treatment by RIE. The insets in (b) and (c) show a magnified image. d) GNDA on an SiO_2_/Si substrate after the PS NSs were removed.

**Figure** **1**a shows the scanning electron microscope (SEM) image of the purchased graphene layer on SiO_2_/Si substrate. Figure 1b shows the SEM image of a closely packed PS NSs layer on graphene on SiO_2_/Si substrate together with its magnified image in the inset, demonstrating the orderly arrangement of the PS NSs over the large area. The diameters of the PS NSs were reduced after O_2_‐plasma treatment by RIE, as shown in Figure 1c and its inset. Figure 1d shows uniformly aligned GNDA on an SiO_2_/Si substrate after the PS NSs were removed.

**Figure 2:**
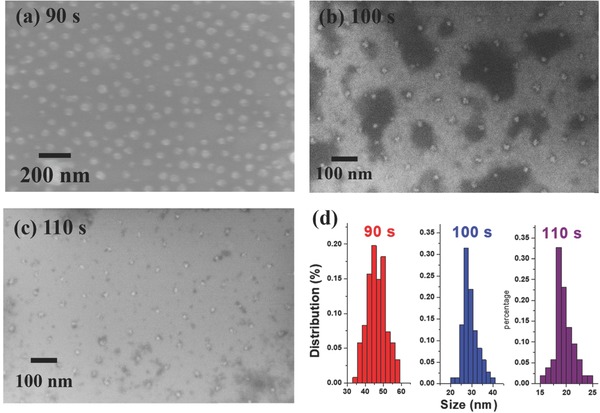
a–c) SEM images of GND arrays for various sizes. The etching time is indicated. d) Size distribution of GNDA.

The SEM images of variously sized GNDAs are shown in **Figure** **2**a–c. When the time of O_2_ plasma treatment was 90, 100, and 110 s, the average size of the GNDA, determined by the size of the remaining PS NSs, reduced to 45, 30, and 20 nm, respectively. The size distributions of three GNDAs are summarized in Figure 2d. The gaps between the GNDs widen as their diameter decreases.

**Figure 3:**
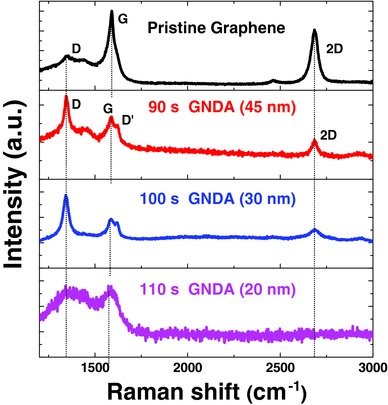
Raman spectra of GNDAs for various sizes and pristine graphene on SiO_2_/Si substrates.

Raman spectroscopy is a powerful nondestructive technique to explore the properties and structure of graphene samples.^18^ **Figure** **3** shows the Raman spectra of the GNDAs for various sizes and pristine graphene on the SiO_2_/Si substrate. The Raman spectrum of the pristine graphene resolves into three distinctive D, G, and 2D bands at ≈1360, ≈1600, and ≈2700 cm^−1^, respectively.^19^ G peak represents a primary in‐plane vibration mode, which is the main characteristic peak of graphene and is quite sensitive to strain. 2D peak stands for a second‐order overtone of a different in‐plane vibration. D band is the disorder vibration peak, which is often employed to characterize its defect or edge.^20^ As graphene changes from a continuous sheet into GNDA, the G and 2D peaks intensity decreased sharply and the D peak intensity increased, indicating the increase of edge carbons. As the RIE treatment process time prolonged, the G and 2D peak intensity of obtained GNDA decreased compared to those of pristine graphene. This change is a strong evidence that the GNDA fabrication process leads to a decrease in the coverage of graphene. Furthermore, the splitting of G peak, so‐called the D′‐band (≈1625 cm^−1^), which is related to the small size and vacancy‐like defect at the edge of graphene,^21^ also supported the increase of edge carbons. The D and 2D bands almost did not shift when the sizes of the GNDAs varied. In comparison, the G band showed a significant size‐dependent shift. The frequency of G band is known to downshift as strain increases. Strain is expected to be more strongly formed at the edge of smaller sized GNDs.

**Figure 4:**
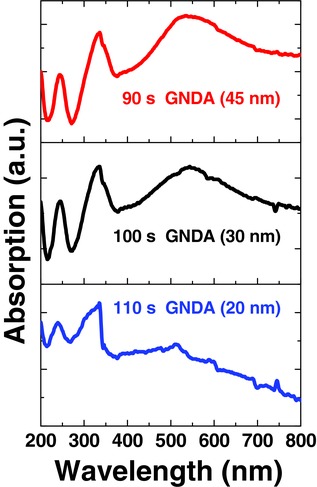
UV–vis absorption spectra of GNDAs with different sizes on SiO_2_/Si substrates.

**Figure** **4** shows the UV–vis absorption spectra of GNDAs with different sizes. The absorption peaks appeared at both UV area and visible area for all GNDAs in this study. The intensity ratio of absorption peak at UV area to visible area increased as GNDA size decreased, which could be attributed to the increasing band gap of graphene dots as size decrease.^22^

### 2.2 Characterization of ZnO/GNDA PD

**Figure 5:**
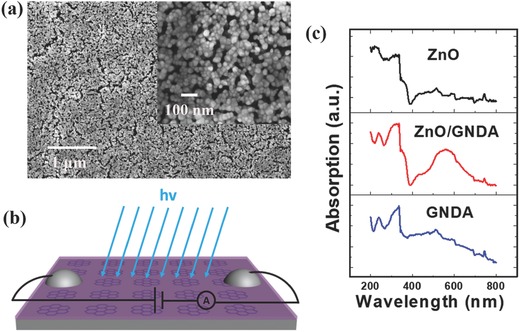
a) SEM image of the ZnO film, the inset shows a magnified image. b) Illustration of ZnO/GNDA photodetector. c) UV–vis absorption spectra of ZnO film, ZnO/GNDA hybrids, and GNDA on SiO_2_ substrates.

The SEM image in **Figure** **5**a of ZnO nanofilm surface shows that it is composed of ZnO nanoparticles (NPs) with diameters of about 20 nm that distributed evenly over the surface. Figure 5c shows the UV–vis absorption spectra of pure ZnO film, ZnO/GNDA composites, and pure GNDA. It can be seen that the ZnO film almost has no absorption in visible area. After combined with GNDA, ZnO/GNDA composites have absorption in both visible area and UV area, which show the absorption characteristics of both pure GNDA and pure ZnO film, indicating the combination of GNDA and ZnO.

### 2.3 Performance Test of Photodetectors

**Figure 6:**
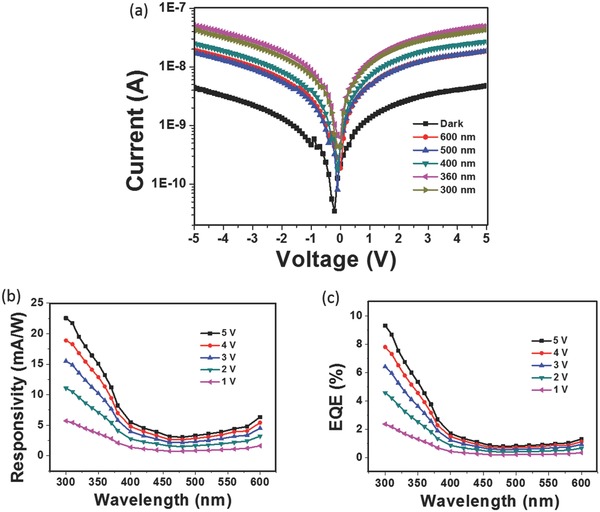
a) *I–V* curves of the ZnO/GNDA (20 nm) PD in dark and under light illumination of various wavelength. b) Responsivity spectra of ZnO/GNDA (20 nm) PD under various biases. c) EQE spectra of ZnO/GNDA (20 nm) PD under various biases.

**Figure** **6**a shows the typical *I–V* characteristics of the ZnO/GNDA (20 nm) PD in dark and under illumination of various wavelengths. The photocurrent was the highest at wavelength of 360 nm among the studied wavelengths. The current was almost the same at the same value of positive voltage and negative voltage, indicating that this photodetector has excellent symmetry to the voltage, which broadens its application under both negative and positive bias.

The photoresponsivity (*R*) spectra of ZnO/GNDA (20 nm) PD under different biases are shown in Figure 6b. Responsivity measures the electrical output per optical input, defined as the ratio of response current to illumination power. *R* = (*I*_Light_ −*I*_Dark_)/(*P*_ill_*S), where *I*_Light_ and *I*_Dark_ are the photocurrent and dark current, respectively, with unit of A, and *P*_ill_ is the incident illumination power, in unit of mW cm^−2^, *S* is the effective illumination area, in unit of cm^2^,^23^ which will be influenced by indium electrodes area in this work. For example, *I*_Light_ was 4.33 × 10^−8^ A at 300 nm at biases of 5 V and *I*_Dark_ was 4.7904 × 10^−9^ A, then the related *R* was 22.55 mA W^−1^ with *P*_ill_ of 0.403 mW cm^−2^ and S of 0.00424 cm^2^. The value of *R* was much lower in 400–600 nm than in wavelength less than 400 nm and it increased significantly when the wavelength was less than 400 nm, indicating the excellent UV detection performance.

The external quantum efficiency (EQE) reflects the number of electron–hole pairs produced by a single incident photon. Figure 6c shows EQE spectra of ZnO/GNDA (20 nm) PD under different biases. EQE = *R* × 1.24/λ × 100%, where *R* is the sensitivity, in units of mA W^−1^, λ is the wavelength of light, in units of nm.^24^ For example, when *R* was 22.55 mA cm^−2^ at 300 nm at biases of 5 V, the related EQE was 9.32%. Figure 6c shows EQE spectra of ZnO/GNDA PD under different biases, of which the variation trend with wavelength was basically the same with spectra of photoresponsivity.

**Figure 7:**
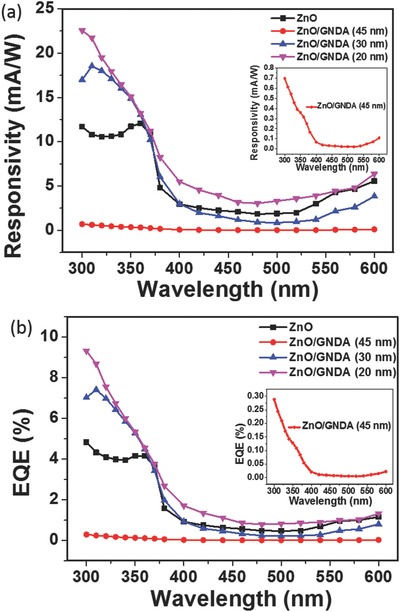
Comparison of photoelectric properties of ZnO PD and ZnO/GNDA PDs with various GNDA sizes. Comparison of a) responsivity and b) external quantum efficiency (EQE) at a bias of 5 V, the inset shows a magnified image of ZnO/GNDA (45 nm) PD.

As mentioned before, the size of GNDA has great influence on its optoelectronic properties, which would lead to different photodetection performance of ZnO/GNDA composites. It is illustrated in **Figure** **7**a that three different fabricated ZnO/GNDA PDs have different responsivity spectra, with comparison of benchmark ZnO PD. Responsivity of both ZnO/GNDA (20 nm) PD and ZnO/GNDA (30 nm) PD appeared significantly larger value than ZnO PD in the range of 360–300 nm. Both ZnO/GNDA PDs had much better responsivity in UV wavelength range of 400–300 nm than in 600–400 nm, indicating good UV light detection sensitivity. As to ZnO/GNDA (45 nm) PD, the responsivity value was much smaller than ZnO PD during the whole studied wavelength range, which could be related to the energy band alignment of heterojunction in ZnO/GNDA (45 nm) PD and the detailed mechanism will be discussed later. EQE of three kinds of ZnO/GNDA PDs show almost similar spectra with responsivity in Figure 7b.

**Figure 8:**
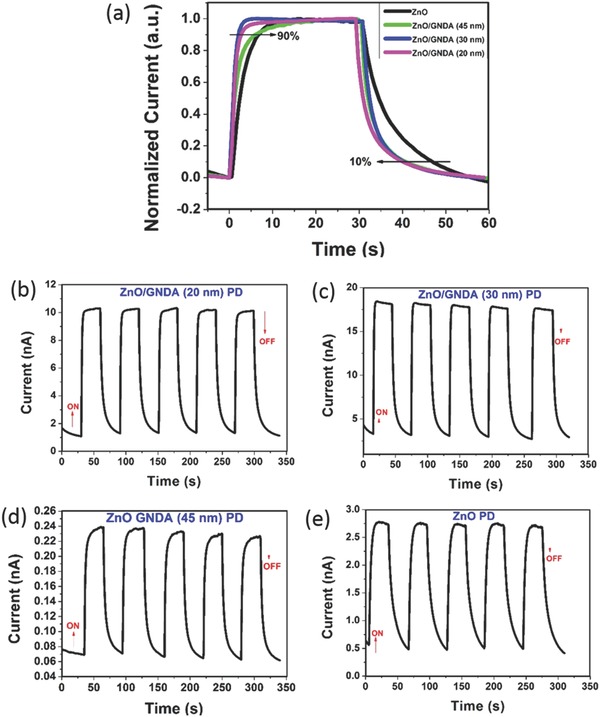
a) Comparison of rise/decay times of fabricated PDs under 360 nm UV light illumination at a bias of 1 V. On–off switching properties of b) ZnO/GNDA (20 nm) PD, c) ZnO/GNDA (30 nm) PD, d) ZnO/GNDA (45 nm) PD, and e) ZnO PD. ZnO/GNDA (20 nm) PD has the largest on/off ratio, resulting its largest responsivity and EQE value among the four PDs.

**Figure** **8**a compares the normalized time‐dependent photocurrents under 360 nm UV light illumination at a fixed bias of 1 V. When the ZnO PD was illuminated, the current increased. However, its current rose slowly upon the illumination and the current decay process lasted for a long time after turning off the UV light: the rise time and decay time were 5.9 and 16.1 s, respectively (the rise/decay time was defined as the time for the current to rise to 90%/decay to 10% of the peak value^25^). In comparison, all the three ZnO/GNDA PDs had a shorter decay time (10.1 s for ZnO/GNDA (45 nm) PD, 9.5 s for ZnO/GNDA (30 nm) PD, 11 s for ZnO/GNDA (20 nm) PD). Besides, both ZnO/GNDA (30 nm) PD and ZnO/GNDA (20 nm) PD appeared much faster response to light on switch and had shorter rise time of 1.9 and 2.5 s, respectively. The improvement could be induced by the heterojunction formation in ZnO/GNDA composites. Figure 8b–e presents the on/off switching characteristics (under 360 nm UV light illumination at a bias of 1 V) of the four devices. The on/off time duration was 30/30 s. It can be clearly seen that all these devices could be switched on/off repeatedly and the results were consistent with what is observed in Figures 6a and 7a.

### 2.4 Mechanisms

For the photodetection differences in four PDs observed in this study, the mechanisms could be related to different band alignment types of heterojunction in ZnO/GNDA hybrids. The reported valence band (*E*_v_) position of ZnO film is from −7.5 to −7.8 eV, and conduction band (*E*_c_) is from −4.1 to −4.4 eV.[[qv: 1a,26]] Many researches have been conducted to explore the band gap of graphene dots.^22^, ^27^ It is reported that the energy gap of graphene dots between the highest occupied molecular orbital (HOMO) and the lowest unoccupied molecular orbital (LUMO) decreases as the size of the graphene dots increases, as evidenced by the theoretical calculation by utilizing tight‐binding method^22^, ^28^ and experimental measurement by UV photoelectron yield spectroscopy (PYS)^6^ and electrochemical method.[[qv: 12,26d]] The energy gap in graphene dots can be tuned over a wide range of wavelengths from the ultraviolet through the visible into the infrared.^8^, ^28^, ^29^ It is determined that the HOMO level of the graphene dots is varied from −5.0 to −5.4 eV by PYS^6^ and the energy band gap can change from 1.6 to 0.5 eV.^28^ According to the reported HOMO level and energy gap, it can be deduced that the LUMO level of graphene dots could vary from −3.4 to −4.9 eV. The HOMO and LUMO shift to higher and lower energies, respectively, as the size of the dot increases.^22^, ^28^ The calculated energy gaps for these graphene dots are inversely proportional to the edge length (*L*) of them,^22^ which is consistent with the UV–vis spectra in Figure 4.

**Scheme 3:**
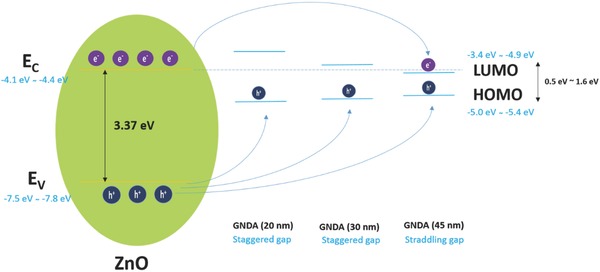
Schematic illustration of the energy band alignment of in ZnO/GNDA hybrids heterojunction.

According to these reported energy level values of ZnO and graphene dots, the HOMO level of graphene dots is between *E*_v_ and *E*_c_ of ZnO, while the LUMO level could be higher or lower than *E*_c_ of ZnO depending on the graphene dots size. As discussed above, the energy band alignment of heterojunction in ZnO/GNDA PDs could be illustrated as **Scheme** **3**. It can be deduced that staggered gap type could exist in both ZnO/GNDA (20 nm) and ZnO/GNDA (30 nm) heterojunction, while ZnO/GNDA (45 nm) could have the straddling gap. Different band alignment types could result different carrier‐transfer processes from the ZnO NPs to the GNDA. Upon UV illumination, photogenerated holes in valence band of ZnO can transfer to HOMO in GNDA, leading to the separation of electrons and holes in ZnO/GNDA (30 nm) PD and ZnO/GNDA (20 nm) PD. Therefore, higher photocurrent, higher responsivity and EQE are achieved in ZnO/GNDA (30 nm) PD and ZnO/GNDA (20 nm) PD compared with ZnO PD. On the contrary, electrons could transfer from *E*_c_ to LUMO in the straddling gap of ZnO/GNDA (45 nm) PD, so the photogenerated electrons and holes have more chance to combine, which results in lower photocurrent and lower responsivity and EQE of ZnO/GNDA (45 nm) PD comparable to ZnO PD.

ZnO/GNDA PDs have the faster response to light on/off switch than ZnO PD, the possible reason may be the formation of heterojunction in ZnO/GNDA PDs. For the ZnO NPs, it is generally accepted that light on/off response is caused by a low‐conductivity depletion layer near the surface, which is related to a very slow oxygen molecules adsorption and desorption processes on the ZnO surface, so the device rise time and decay time are very long.^30^ In the presence of the GNDA, heterojunction forms at the ZnO NPs/GNDA interface. The increase/decrease of the heterojunction depletion layer width is fast under on/off switching light, leading to the short photocurrent rise time and decay time.^31^

## 3 Conclusions

In summary, uniformly sized and aligned graphene nanodots arrays have been fabricated by PS‐NS lithography directly from large‐scale graphene. The GNDA size could be well controlled by O_2_‐plasma treatment time during RIE process from 45 to 20 nm. ZnO nanofilm had been introduced on the surface of GNDA with different sizes through spin coating and annealing to fabricate ZnO/GNDA composites UV PDs. The responsivity spectra and EQE of ZnO/GNDA (20 nm) PD could reach as high as 22.55 mA W^−1^ and 9.32%, almost twofold of that of ZnO PD. Both ZnO/GNDA (20 nm) PD and ZnO/GNDA (30 nm) PD appeared much faster response to light on/off switch and had shorter rise/decay time compared with ZnO PD. However, as the size of GNDA increased to 45 nm, the PD exhibited poor performance, which could be attributed to the straddling gap type of energy band alignment in ZnO and GNDA (45 nm) heterojunction. Our study revealed that the photoelectric properties of ZnO/GNDA were highly GNDA size‐dependent. These efforts have important implications for the understanding of graphene nanodots and exploring the various remarkable photodetection performance by controlling the size of the nanostructures.

## 4 Experimental Section

*Materials*: Monodisperse PS NSs (mass fraction of 10 wt%, average diameter of 100 nm, coefficient of variation less than 5%, bought from Suzhou Zhiwei Nano Technology Co., Ltd., product name S‐PS100), ethanol (analytical grade), deionized water, hexane (analytical grade), sodium dodecyl sulfonate (Sigma‐Aldrich), graphene (on SiO_2_/Si wafer substrate, bought from Suzhou Hengqiu graphene Technology Co., Ltd., coverage ratio greater than 97%, model GR‐SiO_2_/Si‐1‐1), silicon wafer (surface with an oxide layer with thickness of 300 nm), zinc acetate dihydrate ((Zn(CH_3_COO)_2_·2H_2_O), analytical grade), and poly(vinyl alcohol) (PVA, 1788 low viscosity type) were used.

*Fabrication of Size‐Controlled GNDA*: Scheme 1 schematically illustrates the fabrication procedures of GNDA based on PS‐NS lithography. First, a closely packed PS NSs layer was assembled to have sixfold symmetry on the graphene/silicon wafer substrates. For this, 4.5 wt% PS NSs of 100 nm diameter in deionized water were mixed with same volume of ethanol. The mixed solution was ultrasonic treated for 2 h and then was dropped onto graphene by spin‐coating method, the PS NSs solution was spun at 360 rpm for 9 s first and then at 5000 rpm for 40 s. Then RIE was employed to fabricate graphene nanodots array with PS NS layer as mask. During RIE, the graphene exposed to the O_2_ plasma and PS NSs were etched by O_2_ plasma, forming volatile substances to be removed. The size of remaining PS NSs reduced as the etching time prolonged. As a result, only graphene underneath the remaining PS NSs was left. With the ordered PS NS layer as etching mask, an ordered graphene nanodot array formed underneath PS NS layer. During the treatment process, the working pressure, the O_2_ flow rate, and the plasma power were set at 50 mTorr, 30 standard cubic centimeter per minute(sccm), and 50 W, respectively. The diameter of the GNDs was controlled by varying the etching time from 90 to 110 s. To remove PS NSs selectively, the prepared samples were washed out with acetone for 8 h and orderly aligned GNDA were obtained.

*Fabrication of ZnO/GNDA Photodetector*: Zinc acetate dihydrate (Zn(CH_3_COO)_2_·2H_2_O, 2.00 g), PVA (1.00 g), and glacial acetic acid (0.12 mL) were added into deionized water (10 mL). The mixture was heated at 80 °C under constant stirring for dissolution. The prepared solution was then dropped onto the GNDA on SiO_2_/Si wafer and spin coated at 750 rpm for 6 s, then 5000 rpm for 30 s. The prepared film was annealed at 550 °C for 2 h to remove PVA, thus the ZnO/GNDA composites were obtained. The ZnO/GNDA composites photodetector was then constructed using indium electrodes on both sides of ZnO/GNDA composites. The distance between two indium electrodes and the diameter of electrodes were measured by optical microscope. The effective illumination area equals to the distance between two electrodes multiplied by the average diameter of two electrodes. At the same time, pure ZnO photodetector was also fabricated on SiO_2_/Si wafer without GNDA for comparison. Scheme 2 shows the illustration of fabricated ZnO and ZnO/GNDA photodetectors.

*Characterization*: The surface morphologies of the samples were observed with a field emission scanning electron microscope (Zeiss Sigma) with operating voltage of 5 KV. Raman spectroscopy (HR Evolution, France Horiba Jobin Yvon) with an excitation wavelength of 532 nm was used to characterize the properties of the GNDAs and pristine graphene. The absorption spectra were measured using Hitachi U3900H UV–vis spectrophotometer scanning from 800 to 200 nm at a scanning rate of 120 nm min^−1^.

*Photoelectric Measurements*: The photoelectric properties (*I–V* and *I*–*t* characteristics) and spectral photoresponses of the photodetectors were analyzed with an Xe lamp, monochromator, a program‐controlled semiconductor characterization system (Keithley 4200‐SCS, USA). The light intensity was measured with a NOVA II power meter (OPHIR photonics). All the measurements were performed at room temperature.

## Conflict of Interest

The authors declare no conflict of interest.
